# New Appliances and Things Medical

**Published:** 1901-09-21

**Authors:** 


### NEW APPLIANCES AND THINGS MEDICAL.

[We shall be glad to receive, at our Office, 28 & 29 Southampton Street Strand, London, W.C., from the manufacturers, specimens of all new preparations and appliances which may be brought out from time to time.]

### THE "OPEN-AIR" SANATORIUM CHART.

(REYNOLDS AND BRANSON, LTD., 13 BRIGGATE, LEEDS.)

DR. J. M. BARBOUR, of the Swiss-Villa Sanatorium, Swanage, has devised a combined chart on which to note the many details which have to be recorded in regard to patients undergoing sanatorium treatment. Spaces are provided in this chart for temperature and weight; for the amount, character, and presence of tubercle bacilli; for cough, night sweats, diarrhœa, and homoptysis; and for details as to the urine and other excreta. It thus fairly covers the clinical field so far as these cases are concerned. Perhaps the details as to the food taken are intended to go under the head of "remarks"; otherwise we should have thought that some indication of the amount of food and exercise would have been instructive. But we have no doubt that, as they are, these charts will serve a good purpose.

